# Biochemistry of Terpenes and Recent Advances in Plant Protection

**DOI:** 10.3390/ijms22115710

**Published:** 2021-05-27

**Authors:** Vincent Ninkuu, Lin Zhang, Jianpei Yan, Zhenchao Fu, Tengfeng Yang, Hongmei Zeng

**Affiliations:** State Key Laboratory for Biology of Plant Diseases and Insect Pests, Institute of Plant Protection, Chinese Academy of Agricultural Sciences (IPP_CAAS), Beijing 100193, China; 2019y90100111@caas.cn (V.N.); zhanglin42@163.com (L.Z.); jianpei_yan@foxmail.com (J.Y.); 82101185105@caas.cn (Z.F.); yangtengfeng123@163.com (T.Y.)

**Keywords:** terpenes, biosynthesis, phytoalexin, insecticidal, allelopathy

## Abstract

Biodiversity is adversely affected by the growing levels of synthetic chemicals released into the environment due to agricultural activities. This has been the driving force for embracing sustainable agriculture. Plant secondary metabolites offer promising alternatives for protecting plants against microbes, feeding herbivores, and weeds. Terpenes are the largest among PSMs and have been extensively studied for their potential as antimicrobial, insecticidal, and weed control agents. They also attract natural enemies of pests and beneficial insects, such as pollinators and dispersers. However, most of these research findings are shelved and fail to pass beyond the laboratory and greenhouse stages. This review provides an overview of terpenes, types, biosynthesis, and their roles in protecting plants against microbial pathogens, insect pests, and weeds to rekindle the debate on using terpenes for the development of environmentally friendly biopesticides and herbicides.

## 1. Introduction

Plants and a multitude of pathogenic microbes are in a constant battle for supremacy. While pathogens adopt novel means to maintain their nutrition and shelter sources in plants, the former undergoes immunity evolution to keep off the latter by eliciting defense molecules. Despite the absence of motile defensive cells and adaptive immunity, plants possess a robust immune system with a mirage of defense molecules to enhance their physical and chemical immunity against environmental stresses [1]. Plants produce two types of metabolites; primary metabolites are involved in cellular survival and propagation, and secondary metabolites play a crucial role in defense against pathogens and pests. Plants synthesize over 300,000 secondary metabolites, and muting or silencing their synthesis severely impairs their effectiveness to withstand biotic stresses [2].

PSMs are categorized as phenolics, terpenoids, and alkaloids, among others. They play several roles in plant defense against fungi, viruses, bacteria, and feeding herbivores [3]. Some PSMs perform allelopathic activities, while others serve as signal transducers for chemical communication with symbiotic insects, such as pollinators and seed dispersers [4]. The utilization of PSMs for pharmacological purposes spanned several thousands of years. However, analysis of PSMs for specific functional annotation came into lamplight only a couple of hundred years ago, with morphine from *Papaver somniferum* (opium poppy) among the earliest studied. Several PSMs have recently been investigated for pesticides/insecticidal and herbicidal activities. Approximately 40% of commercial medicines are synthesized using PSMs as the active ingredients [5,6]. For example, Taxol^®^, an anticancer drug, and artemisinin, an antimalarial drug, are synthesized from terpenes [7]. Diterpenes and phenylpropanoids secretion, for instance, confer phytoalexin properties against pathogenic microbes [8]. Antifeedant metabolite synthesis also enhances plant resistance to herbivores. For example, alkaloids deter *Empoasca fabae* and *Leptinotarsa decemlineata* [9], and phenolic compounds repel *Rhopalosiphum padi* and *Galerucella lineola* from feeding on wheat plants and also delay maturation and lower fecundity in *Aphis gossypii* on cotton plants [10].

Terpenes are the largest and most diverse PSMs in nature [11,12] and are the informative and defensive vehicles used by plants for antagonistic and mutualistic interactions. The production of terpenes by plants to counter biotic (pathogenic microbes, herbivore pests, and weeds) and abiotic (water, temperature, light, and salt) stresses is widely studied [8,13,14,15]. While phytoanticipins terpenes are constitutively secreted in the absence of pathogen effectors, phytoalexins are elicited in response to inducible pathogenic microbes or feeding herbivores [7]. Terpene research as an active ingredient in pesticide development has seen a global resurgence due to its low risk to the environment and human health. For example, a consistent but deliberate policy in the European Union reduced the number of permissible synthetic compounds from 1000 in 1993 to 250 in 2011. The shift from risk to a hazard-based assessment of the effect of synthetic pesticides on humans and the environment accounted for this reduction. Costs for outdooring a new synthetic active product hiked amidst overwhelming cases of pesticide resistance, as Sparks and Nauen, 2015 reported that over 580 species of arthropods globally developed resistance to synthetic pesticides [16,17].

The crucial role of PSMs and terpenes makes them a critical topic of concern to plant protectionists. This write-up reviews established knowledge of terpenes, types, the chemistry of their biosynthesis, and function in protecting plants from pests, diseases, and weeds. These highlights will reinvigorate the debate on using terpenes as active ingredients in developing biopesticides.

### 1.1. General Overview of Terpenes

The term terpene, proposed by Dumas in 1866, originates from the Latin word ‘turpentine’ (*Balsamum terebinthinae*), a liquid extract from pine trees. Terpenes are the largest natural products, with significant structural variation, including linear hydrocarbons or carbocyclic skeletons. Approximately 55,000 members are known [18,19]. Terpenes undergo oxygenation, hydrogenation, or dehydrogenation to form terpenoids. Terpenes classification is based on Wallach’s, 1887 proposed isoprene units (C5H8), a 5-carbon compound that forms terpenes backbones [20,21]. The isopentenyl diphosphate (IPP) unit and its isomer, dimethylallyl diphosphate (DMAPP), are the biosynthesis precursors of terpenes. Terpenes are abundant in higher plants, citrus, conifers, and eucalyptus and are widely distributed in the leaves, flowers, stems, and roots of these plants. The development of chromatographic and spectroscopic methodologies in 1945 propelled the explosive discovery of terpenoids and terpene-derived products. Terpenoidal molecules are antifungal, antimicrobial, antiviral, and antiparasitic. They deter feeding herbivores and are used as insecticides to store agricultural products [18]. However, their full potential has not yet been attained.

### 1.2. Classification of Terpenes 

The isoprene unit that defines terpene consists of a head and a tail. According to Ingold, terpenoids bond by head-tail linkage of isoprene units. Tetraterpenes, such as carotenoids, deviate from this rule by centrally forming a tail–tail (4-4) bond [18,19]. Some terpenes have high vapor content and are therefore classified as volatile (VTs), e.g., hemiterpenes, monoterpenes, and sesquiterpenes. Others are semi-volatile or non-volatile, such as diterpenes. The number of isoprene units on a terpene backbone generally accounts for their volatility. Fewer isoprene units are highly volatile terpenes [22]. 

**Figure 1:**
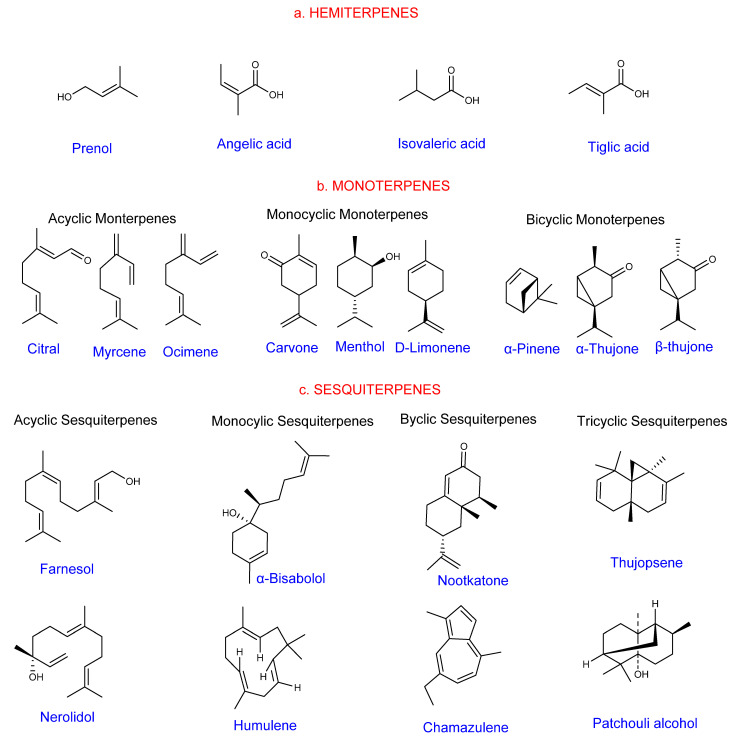
Structural forms of volatile terpenes (structures were drawn and analyzed with ChemDraw software, version 20.0.0.41). (**a**) Hemiterpenes; (**b**) Monoterpenes; (**c**) Sesquiterpenes.

Hemiterpenes are the simplest, with a single isoprene unit. The oxygen-containing derivatives of the isoprene unit form other hemiterpenes (Figure 1a). Hemiterpenes are being investigated as potential sources of biofuel [18].

Monoterpenes are highly diverse and occur in monocotyledonous and dicotyledonous angiosperms, fungi, bacteria, and gymnosperms. They have two isoprenoid units [23]. They are odoriferous compounds that partly account for the scent of many flowers and fruits. Approximately 18 rice monoterpenoids play varying roles, including defense against pathogens and pests [24]. Monoterpenes include acyclic, monocyclic, and bicyclic forms (Figure 1b). They are components of essential oil compounds that give plants aroma and flavor and are vital for a range of active ingredients for agricultural, pharmaceutical, cosmetic, and food applications. Pinenes, carveol, camphor, menthol, and limonene, for example, are active ingredients in a variety of industrial applications [25]. 

Sesquiterpene is an abundant natural compound with 3-isoprene [26]. They share ring classification with monoterpenes, an exception being a few tricyclic terpenes (Figure 1c). This terpene diversity arises from the arrangement of the 15-carbon skeletons, the layering of the functional groups, and the substituents on their backbone [27]. Some members are hydrocarbons (humulene, farnesene), aldehydes (farnesal and lepidozenal), oxygenated hydroxyl or carbonyl derivatives, and esters (torilin and ejaponines). Some are also alcohols, such as δ-elemanol and β-germacrenol [28]. They are antimicrobial, antifungal, antitumor, and anti-inflammatory agents. They have wide plant defense applications against herbivores and are active constituents in the perfumery industry [29]. However, these potentials have largely remained unexplored in the biotechnology industry. 

**Figure 2:**
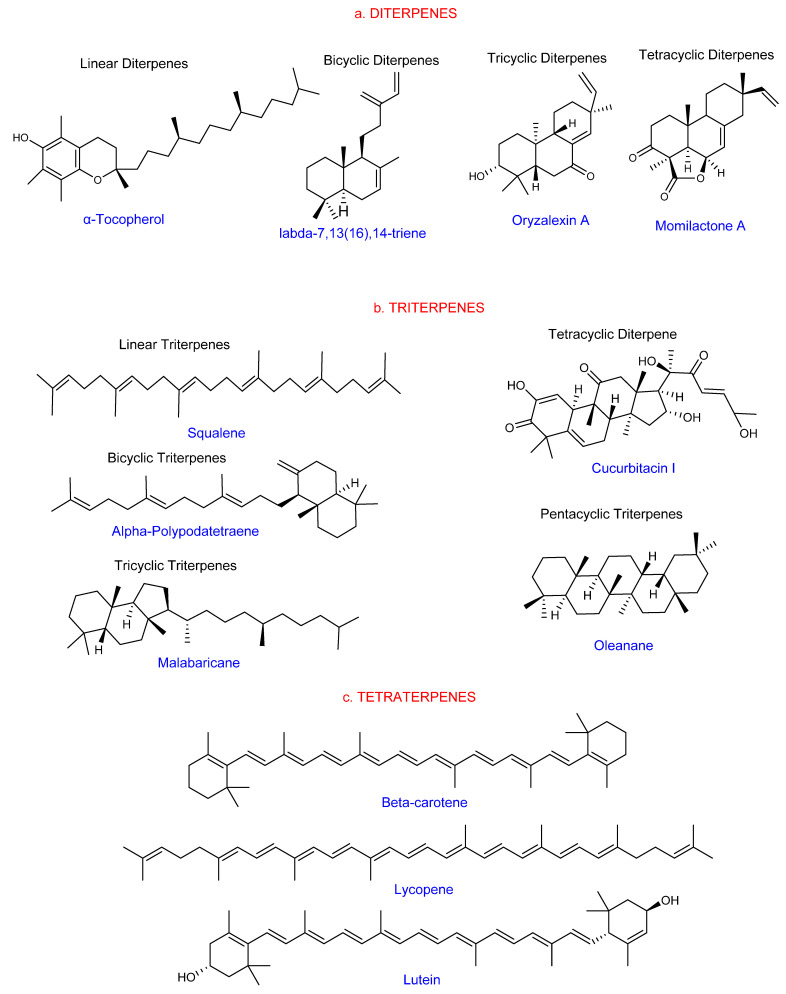
Structural forms of semi and non-volatile terpenes (structures were drawn and analyzed with ChemDraw software, version 20.0.0.41). (**a**) Hemiterpenes; (**b**) Monoterpenes; (**c**) Sesquiterpenes.

Diterpenes are non-volatile C20 hydrocarbons derived from four isoprene units and are structurally diverse [18,30]. They include linear, bicyclic, tetracyclic, pentacyclic, or macrocyclic forms (Figure 2a). Diterpenes are characterized by ployoxygenated keto and hydroxyl groups [26]. Diterpenes from various sources have exhibited inhibitory effects against pathogenic microbes, herbivore pests, and weeds. These promising biological activities place them among the essential agricultural secondary metabolites with potential in the production of biopestides [31]. Rice plants produce several phytoalexins and allelochemical diterpenes for protection against pathogens, pests, and weeds. The accumulation of these compounds, on the other hand, is low and can be increased by genetic alteration or the recruitment of exogenous elicitors.

Triterpenes are derivatives of the C30 precursor, squalene, with over 20,000 known members. Most are members of the plant kingdom. However, bacteria and sea cucumbers produce defense-related triterpene glycosides [14]. Two sesquiterpene molecules form triterpenes by linking in a head–head fashion [32]. Cyclic triterpenes (1–5 rings) are the most significant members. They are primarily alcohols, aldehydes, or carboxylic acids [27]. A cyclopentane perhydrophenanthrene ring system defines sterols and phytosterols as triterpenes [32]. Glycosylated triterpenes, such as saponins, protect plants against pathogenic microbes and insect pests. Some simple triterpenes are signaling molecules that are also constituent ingredients in the food, health, and biotechnology industries. [14]. The structures of triterpenes are shown in Figure 2b.

Tetraterpenes (carotenoids) are 8-isoprene units consisting of C40 and C_40_H_64_ molecular formulas [33]. Carotenoids are the most studied tetraterpenes, with more than 750 members [34]. Terrestrial plants, algae, and cyanobacteria all produce tetraterpenes. Their biological roles include light trapping, antioxidative function, and plant protection against free radicals. They are also involved in plant hormone synthesis and form the structural components of cell membranes. They are active ingredients in the pharmaceutical and food industries [35,36]. The structures of the tetraterpenes are shown in Figure 2c.

## 2. Biosynthesis of Terpenes 

**Figure 3:**
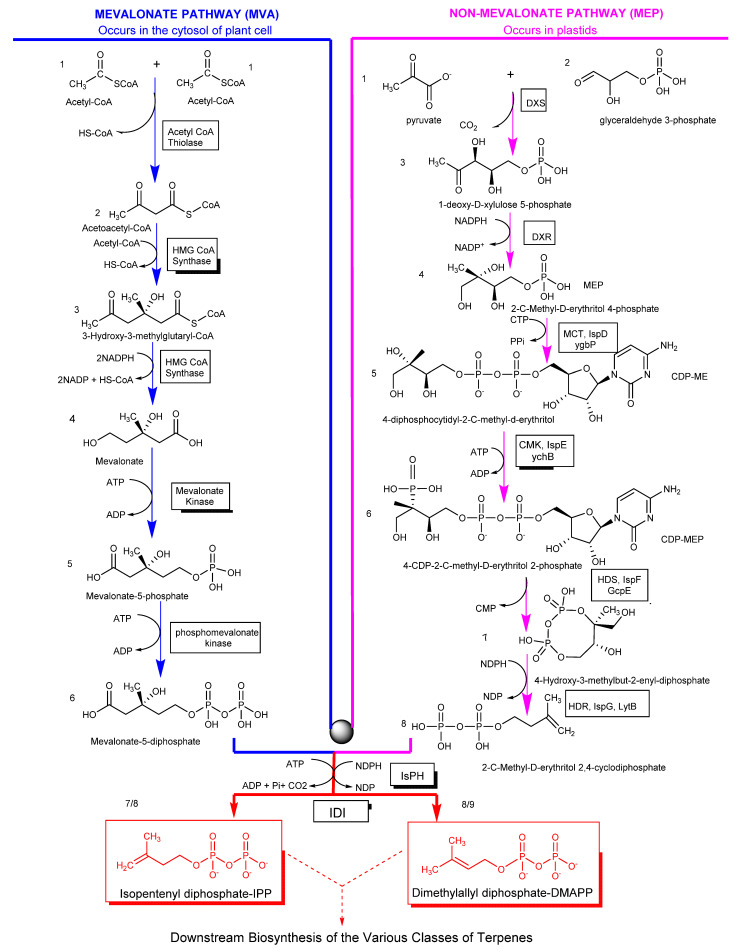
Biosynthesis of isoprenoid precursors (IPP and DMAPP) [40,41,42,43,44,45] (structures were drawn and analyzed with ChemDraw software, version 20.0.0.41).

Despite being widely accepted as the basic unit of terpenes, terpenes synthesis is not initiated via the isoprene unit. Two distinct molecules with a similar structural arrangement as the isoprene unit (isopentenyl diphosphate (IPP) and dimethylallyl diphosphate (DMAPP)) are the precursors for the synthesis of terpenes. These molecules are the products of two independent pathways. The mevalonate pathway (MVA), discovered in the 1950s by Lynen, Bloch, and Cornforth, occurs in animals, fungi, cytosols of plants, archaea, and a few bacteria. Furthermore, the 2C-methyl-D-erythritol-4-phosphate (MEP) pathway, discovered by Lichtenthaler, Rohmer, Arigoni, and Seto in the 1990s/2000s, occurs in the plastids of plants, green algae, and most bacteria. Both IPP and DMAPP subsequently undergo rearrangements, repetition, and cyclization reactions to yield various terpene classes [37,38,39]. Despite variations in the start-up molecules in the two pathways, the same IPP and DMAPP are formed (Figure 3).

**Figure 4:**
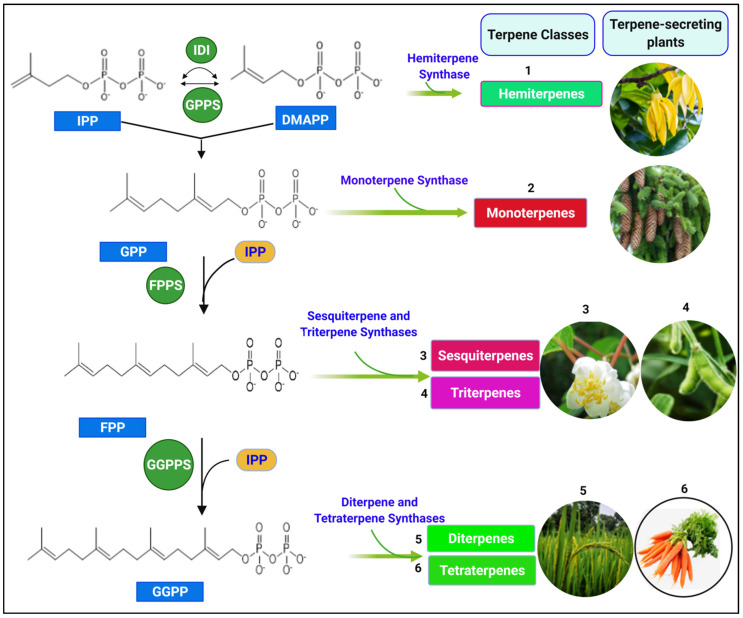
Biosynthesis of various classes of terpenes from IPP and DMAPP (structures were drawn and analyzed with ChemDraw software, version 20.0.0.41, and uploaded on BioRender.com to create the illustration).

IPP and DMAPP undergo a condensation reaction, catalyzed by geranyl pyrophosphate synthase (GPPS) and farnesyl pyrophosphate synthase (FPPS) to produce GPP (C10_monoterpene) and FPP (C15_Sesquiterpenes). Geranylgeranyl pyrophosphate synthase (GGPPS) and farnesyl geranyl pyrophosphate synthase (FGPPS) catalyze a similar enzymatic reaction to produce GGPP (C20_diterpenes) and FGPP (C25_sesterpenes). The terpene synthase family further catalyzes GPP, FPP, GGPP, and FGPP precursors cyclization, rearrangement reactions to form various terpenoid classes (Figure 4) [40,41,42,43,44,45]. 

### 2.1. Terpene Synthases (TPSs), Classes, and Terpenes Encoded 

The structural diversity of terpenes is due to the broad terpene synthases superfamily (TPSs). Over a hundred TPS genes and prenyltransferases are known [46,47]. Some of the enzymes involved in the post-cyclization alteration of terpenes include methyltransferases, P450s, and NAD+-dependent dehydrogenases [48].

TPS of gymnosperm origin is different from angiosperms. The entire TPS length, according to the Pfam database, includes N-terminal and C-terminal domains. While the N-terminal has a single motif, the C-terminal has two aspartate-rich motifs [49]. DDxxD and NSE/DTE motifs on the C-terminal domain are responsible for divalent ion(s) coordination and stabilization of water molecules on the active site [49]. The length of TPS varies depending on the terpene class. Generally, monoterpenes synthases are 600–650 amino acid residues long. Sesquiterpenes are 550–580 amino acid residues long, and about 380–440 amino acid lengths are found in diterpenes. TPSs were initially grouped into six subfamilies based on their amino acids full-length or whether they are angiosperms- or gymnosperms-based. TPS sub-families arising from gymnosperm and angiosperm clads are both species-specific and non-species-specific. For example, in Arabidopsis, 10 genes encoding the synthesis of sesquiterpenes and the putative diterpene synthases are close relatives compared to those in other angiosperms. Among the two TPSs families (Class I and II), the subfamilies including TPSa, TPSb, TPSc, TPSd, TPSe, and TPSf are known. However, TPSf and TPSe were recently merged as TPSe/f because they originate from the same ancestral clad, and also, TPSf is a derivative of TPSe. Moreover, TPSg and TPSh from Angiosperms and *Selaginella moellendorffii* have recently been discovered [50,51,52].

The advent of the next-generation sequencing tool paved the way for the transcriptional studies of many plant genomes, including rice, maize, Arabidopsis, and tomato, resulting in the revelation of enormous functionally elucidated terpene synthase families. The Arabidopsis genome encodes 32 full-length TPSs. Twenty-two of which belong to the TPSa subfamily, and 6 are members of the TPSb subfamily. One each belongs to TPSc and TPSg subfamilies, respectively. The remaining two are from the TPSg subfamily. A total of 34 and 24 TPS subfamilies have been identified in *Oryza sativa* and *Sorghum bicolor*, respectively. Furthermore, *Solanum Lycopersicum* (tomato), *Populus trichocarpa* (black cottonwood), and *S. moellendorffii* genomes contain 44, 32, and 14 TPSs, respectively [52,53,54].

TPSa members have no conserved arginine/tryptophan motif RRX8W. They encode only sesquiterpene synthases in dicot and monocot plants. Contrary to TPSa, TPSb and TPSg are angiosperm-specific subfamilies. TPSb has a conserved R(R)X8W motif and is responsible for launching isomeric cyclization reactions. The TPSg subfamily lacks the R(R)X8W motif in its coding protein and plays a role in the biosynthesis of acyclic monoterpenes that comprise volatile organic compounds (VOCs) [53,55,56]. Some members of the TPSg subfamily produce acyclic sesqui- and diterpene products aside from monoterpenes [52]. The subfamily TPSc, identified with the DXDD motif instead of DDXXD, is primarily present in terrestrial plants. TPSd is gymnosperm-specific and encodes mono-, sesqui-, and di-terpene synthases. Furthermore, TPSe/f subfamily members are mainly found in vascular plants, encoding kaurene synthases, and copalyl diphosphate synthases. They function in the biosynthesis of gibberellin. The TPSh subfamily is the sole precursor to *S. moellendorffii*, capable of encoding DXDD and DDXXD motifs [52].

### 2.2. Elicitor-Induced Terpenes Biosynthesis

Elicitors are molecules that trigger responses in plants. They are chemically diverse and act in a broad-spectrum manner [57]. Their renaissance has heightened the study of plant immunity. They are a possible remedy for biopesticides when recruited as external stimuli to induce transient accumulation of defense molecules in plants. Both biotic and abiotic elicitors induce terpene biosynthesis. Biotic elicitors are molecules derived from living organisms such as fungi, bacterial, and cell wall fragments of plants. Abiotic elicitors are physical and chemical-related stresses with derivatives such as organic or inorganic compounds, salt, and heat stresses [58,59]. While plants produce enough terpenes for their protection, inducing up-regulated production will meet commercial quantities for biopesticide development [58,60].

The advent of sophisticated whole-genome sequencing tools, such as Next-generation Sequencing (NGS) technologies, has demystified the identification of induced gene studies [61]. According to Ma et al., several genes play a role in terpenes biosynthesis [62], and elicitor molecules can induce the high expression of the genes [63]. Elicitors can activate the methylerythritol phosphate (MEP) pathway to synthesize terpenes. Gene expression analysis of rice-cell suspension-culture treated with chitin induced early expression of MEP pathway genes for phytoalexins biosynthesis. In a related report, an HR-inducing elicitor protein, Mohrip1, isolated from *Magnaporthe oryzae*, was implicated by RNAseq and qPCR to induce high expression of oryzalexin genes that eventually compromised *M. oryzae* virulence [63]. Farag et al. also demonstrated that salicylic acid could enhance diterpenes biosynthesis in *Sarcophyton ehrenbergi* [64]. In the last couple of years, transient expression of *N. benthamiana* to reconstitute a partial or complete pathway for the synthesis of natural products has been reported [65,66,67]. This genetic modification has a significant effect on terpene production in higher titers. For example, momilactone diterpenoids induce allelopathy in rice plants. The momilactone gene transiently expressed in *N. benthamiana* successfully exhibited allelopathic activities. Re-routing the diterpene biosynthesis site from the chloroplast to the cytosol was the technique. The reconstituted momilactone biosynthetic pathway synthesized momilactone by over 10-fold. Purified momilactone B from the *N. benthamiana* stifled *Arabidopsis thaliana* germination [68]. Gao and team also revealed that an endophytic fungal elicitor obtained from *Fusarium* sp. E5 induced the accumulation of isoeuphpekinensin and euphol in *Euphorbia pekinensis* suspension cultures by 5.81% and 3.56%, respectively [69].

A couple of research reports have also indicated that *Taxus cuspidate* accumulates following methyl jasmonate (MeJA), hydrogen peroxide, salicylic acid (SA), and fungal elicitor (F3) treatment. These elicitors simultaneously enhanced the activity of 10-deacetylbaccatin III-10-O-acetyltransferase (10-DBAT) and cytochrome P450 monooxygenase concentration for high taxol synthesis [70,71]. These shreds of evidenceindicate that elicitors are vital players in terpene synthesis that could meet commercial quantities. 

## 3. Role of Terpenes in Plant Protection

The field of plant protection involves safeguarding plants against pests, diseases, and weeds. Biological control of pests dates to several years ago. The BIOCAT database reveals various insects and their natural enemies applicable in agriculture [72].

### 3.1. Role of Terpenes in Insect Modulation

Host identification of feeding insects largely depends on either visualization, olfactory cues, or both for chemotactic and landing efficiency. The embodiments of olfactory cues are mostly C_5_, C_10_, and C_15_ isoprene chain terpenes produced in the host plants’ glandular trichomes or epidermis for insect defense. These compounds confer antixenosis, a mechanism that disrupts arthropods’ mode of life or indirectly attracts their natural enemies [73].

Besides gibberellins and brassinosteroids having primary roles in growth and development, most terpenes are defense compounds. Terpenes can be alarm substances, defensive emissions, trail markers, and deterrent antifeedant substances. Terpenes contain over 25,000 VOCs with varying concentrations and toxicity levels used for defense against insects and pathogenic microbes. Plants elicit VOCs under stress conditions. For example, the monoterpene-derived compound, cyclopentanoid (iridoid) is incredibly bitter and a robust defensive agent against insect pests. Iridoid glycosides covalently bond to nucleophiles to form amino acids, proteins, and nucleic acids. This bond causes the denaturation of proteins, amino acids, and nucleic acids. This phenomenon reduces plant nutrients, depriving the insects of proteins and nucleic acids. Iridoids can also inhibit the synthesis of prostaglandins and leukotrienes in insects, thereby stifling their growth and development. Under in vitro conditions, iridoid prolongs the larval stage and reduces insects’ growth and survival rates [74,75,76]. 

Flowers and leaves elicit neurotoxin-modulating monoterpene esters called pyrethroids. This terpene harms wasps, bees, beetles, and moths. Studies have shown that pyrethroid is harmless to the environment and, therefore, used as active ingredients in most commercial insecticides. Methylcyclopentanoid monoterpenes produced by *Teucrium marum* (cat thyme or kitty crack) is a repellent to ants and cockroaches. α-pinene, β-pinene, limonene, and myrcene accumulate in the spindle twigs of conifers are toxic to beetles and most pests of conifers [77,78,79]. Essential oils from Gaultheria (Ericaceae) and Eucalyptus (Myrtaceae) also repel several insects, including the housefly, drugstore beetle, and rice and bean weevils [80]. Moreover, momilactone A, a rice diterpenoid, also prevented white-backed planthopper infestation [81,82].

A sesquiterpene (*E*)-β-caryophyllene accumulated following herbivore’s attack on maize plants. Although (*E*)-β-caryophyllene was not directly involved in the defense against the herbivores, it attracted natural enemies to fight them. (*E*)-β-caryophyllene was synthesized as a decoy to attract entomopathogenic nematodes to fight off the attack from *Diabrotica virgifera virgifera* (corn rootworm) [83]. (E)-β-caryophyllene, in a related study, attracted *Cotesia sesamiae* (larval parasitoid) of *Chilo partellus* (spotted stalk borer). These lepidopteran stem borers are a threat to cereal production as studies showed they could reduce about 80% yield [84]. OsTPS3 and OsTPS13 genes encode (E)-beta-caryophyllene synthase and (E, E)-farnesol synthase, respectively, in rice plants. OsTPS3 protein under in vitro conditions catalyzed the synthesis of (E)-beta-caryophyllene, alpha-humulene, and beta-elements in rice plants. The transgenic lines of OsTPS3 in *Oryza sativa* induced high production of (E)-beta-caryophyllene upon methyl jasmonate (MeJA) treatment. This consequently attracted parasitoid wasps of *Anagrus nilaparvatae* (Hymenoptera: Mymaridae), implicating OsTPS3 in signaling volatile sesquiterpenes [85]. Furthermore, the over-expression of FPS2 in Arabidopsis with chloroplast as a target stimulated the production of E-β-farnesene and other sesquiterpenes that subsequently induced resistance against aphids [86].

The tea green leafhopper is a harmful pest of *Camellia sinensis* (Green tea). β-1,3-glucan laminarin induced elicitation of volatile compounds that reduced the green leafhopper population by attracting the egg parasitoid wasp [87].

**Figure 5:**
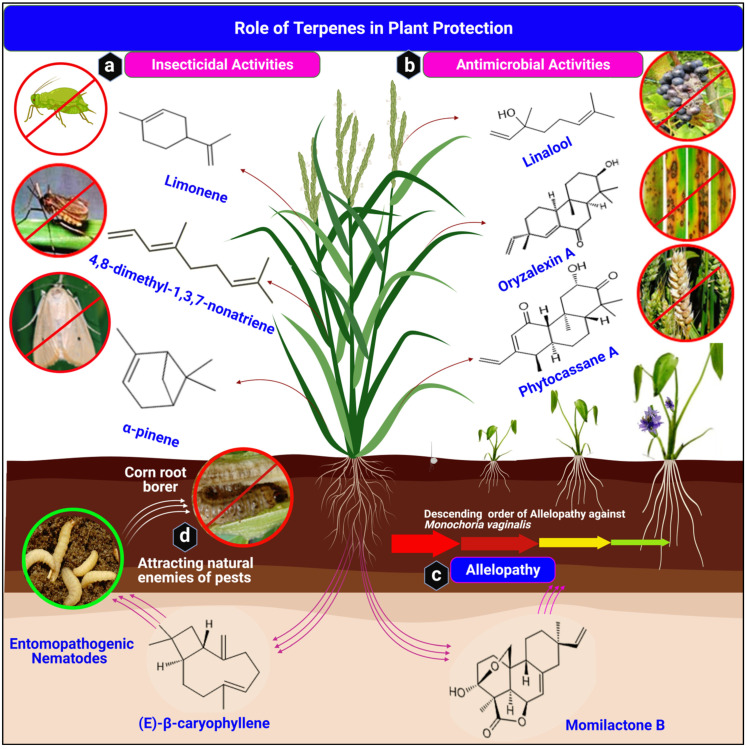
Terpenes’ role in plant protection. Four critical functions of terpenes in plant protection: (**a**) insecticidal role of terpenes, (**b**) antimicrobial activities of terpenes against rice blast disease and *Fusarium* head blight, and (**c**) allelopathy of root exudates of plants. Momilactone B inhibits the growth and development of *Monochoria vaginalis*. (**d**) Terpenes indirectly attract natural enemies of pests, e.g., (E)-β-caryophyllene attracts entomopathogenic nematodes on corn root borer. (Structures were drawn and analyzed with ChemDraw software, version 20.0.0.41, and uploaded on BioRender.com to create the illustration).

Terpene-based biopesticides have been in use for some time now. Some apply active ingredients from orange or citrus oils, essential oils obtained from the *Chenopodium ambrosioides* variety, and neem extracts [88]. An essential oil recently purified from the floral whorls of *Cannabis sativa* L by gas chromatography and gas chromatography-mass spectrometry methods proved effective against some insects. Aphids and mosquito larvae were all killed. A total of 66% of tobacco cutworms and 80% and 33% of houseflies and adult mosquitoes, respectively, failed to survive. The oil composition included 45.4% beta-caryophyllene, 25.0% myrcene, and 17.9% α-pinene-.. There were also 8.3% humulene, 5.2% β-pinene, 5.1% ocimene, and 3.0% of farnesene. Intriguingly, only 3% of beneficial insects, such as ladybug larvae, were killed at the highest concentration [88]. Such a selective modulation nature makes a good starting material in isolation or combination for pesticide development. Figure 5a and Table 1 illustrate the role of terpenes in defense against insects. 

### 3.2. Antimicrobial (Phytoalexin) Activities of Terpenes

There is a substantial amount of literature on phytoalexin terpene research. Phytoalexin camalexin synthesis in *Arabidopsis* defends against *Pletosphaerella cucumerina*, *Botrytis cinerea*, and *Alternaria brassicicola* [89,90]. 

Terpenes are a vital member of the VOCs group of weapons often unleashed by plants to defend against various fungi, bacteria, and viruses. (*E*)-β-caryophyllene is a widespread VOC in the plant kingdom and is produced from the floral whorls. This terpene critically inhibited a rod-shaped Gram-negative bacterium, *Pseudomonas syringae,* on Solanaceae and *Arabidopsis thaliana* plants. In the flowers of *Arabidopsis thaliana*, bacterial growth exponentially increased when the production of (*E*)-β-caryophyllene was muted and critically inhibited and triggered defense signaling pathways when it was restored [91]. Capsidiol is another sesquiterpene consisting of toxic compounds with a significant phytoalexin property. Capsidiol inhibited *Phytophthora capsici* and *Botrytis* *cinerea* virulence in *Nicotiana plumbaginifolia*. Recent molecular evidence in NbEAS or NbEAH-silenced *N. benthamiana* suggests a compromised defense function against *P. infestans* [92] and Potato Virus X (PVX) [13]. Song et al. also revealed that capsidiol inhibited *A. alternata* under in vitro conditions. They argued that the ERF2-like transcriptional factor is responsible for the biosynthesis of capsidiol, and its role as a phytoalexin is independent of ethylene and JA signaling pathways [93].

Diterpenes also play a role in the plant’s immune response against microbial pathogens, particularly *M. oryzae*. For example, the rice plant produces four major labdane-related diterpenoids that function as phytoalexins. They are momilactone A and B, phytocassanes A–F, oryzalexin A–F, and oryzalexin S [94,95]. The identification of these diterpenes was based on their defense activities against rice blast fungi, *M. oryzae*, leaf blight pathogen, and *Xanthomonas oryzae* pv *oryzae* (Xoo) [96]. Diterpene (11E,13E)-labda-11,13-diene-8alpha,15-diol, designated WAF-1, was implicated in immune defense signaling against tobacco mosaic virus (TMV) in *Nicotiana tabacum*. WAF-1 induced salicylic acid protein kinase by activating pathogenesis and wound healing-related genes. The endogenous level of WAF-1 in TMV tobacco leaves increased due to the hypersensitive response (HR) [91,92].

Oryzalexin diterpenoids isolated from rice leaves induced strong resistance to bacterial leaf spot disease. Notable among them are oryzalide-related diterpenes: oryzalic acid A, oryzalide, ent-15,16-epoxy-3β-palmitoyloxy-kauran-2-one, ent-15,16-epoxy-3α-palmitoyloxy-kauran-2-one, ent-15,16-epoxy-3β-hydroxy-kauran-2-one, ent-15,16-epoxy-2,3-dihydroxy-kaurane, and oryzadione ent-15,16-epoxy-kauran-3-one [24]. Following *M. oryzae* infection of rice plants, 9β-pimara-7,15-diene-3β,6β,19-triol, phytocassane F, and stemar-13-en-2α-ol accumulation increased in the leaves. Among these three compounds, phytocassane F showed stronger inhibition of the fungal mycelial growth [97]. Triterpenes are weak phytoalexin compounds. However, quinoa, a rice husk triterpene, exhibited effective molluscicidal and antimicrobial activities [98]. Phytocassanes A–D were also reported to inhibit *M. oryzae* and *R. solani* in rice plants and subsequently hindered the growth of *M. oryzae* under in vitro conditions. A related study confirmed their accumulation in abundance within necrosis sites of the *M. oryzae* infections [99]. Two labdane-related diterpenes, sclareol and cis-abienol, isolated from tobacco, exogenously inhibited bacteria wilt diseases in tobacco, tomato, and Arabidopsis plants. Microarray analysis implicated genes encoding mitogen-activated protein kinase (MAPK) cascade components, ATP-binding cassette (ABC) transporters, and biosynthesis and signaling defense-related molecules [100]. These events showed that host immune factors were responsible for inhibiting wilt disease.

Epoxydolabranol, a carbotricyclic tetradecahydrophenanthrene diterpene elicited in maize roots, effectively inhibited the pathogenesis of *Fusarium graminearum* and *Fusarium verticillioides* simultaneously [101]. Another maize terpenoid, zealexins, is an effective antimicrobial agent. Zealexin A1 resisted the growth of *A. flavus*, *F. graminearum*, and *R. microspores*. Zealexins A3 and A4 also exhibited inhibitory effects against *A. flavus* and *F. graminearum* [37,102,103]. The Antimicrobial properties of terpenes are illustrated in Figure 5b and Table 2.

### 3.3. Allelopathic Activities of Terpenes in Agriculture

Agronomic weeds cause the loss of estimated annual revenue of about $95 billion globally. Weeds have severe effects on crop growth, pest buildup, and nutrient depletion. For example, *Echinochloa crus-galli* is a devastating weed that depletes about 80% of soil nitrogen. It is also a favorable host for mosaic virus diseases. It grows up to 60 inches tall, producing about 40,000 seeds annually. These features make this grass a good competitor in the rice field, reducing yield to about 50% with only 25 members per m^2^ [104,105,106,107,108]. However, synthetic herbicides are an unfavorable antidote to weed control due to their adverse effect on the environment and biodiversity. Exploiting the allelopathic potential of plants, which has been in use since ancient times, remains promising in inhibiting several weeds. Some higher plants are functionally annotated to possess allelopathic effects. *Medicago sativa* L, buckwheat, hairy vetch, and velvet bean, among others, are described as paddy field natural herbicides due to their allelopathy effect [109,110]. Allelochemicals are toxic organic root exudates that can negatively impact the physiological performance of neighboring plants. These chemicals stifle respiration and germination, ion uptake, and photosynthesis of weeds. Stomatal opening, transpiration, enzyme actives, and hormonal levels are also negatively impacted by allelochemicals. Allelopathic activities could also hinder cell division and differentiation, gene expression and signal transduction, and truncate cell membrane permeability [111]. 

Monoterpenes, sesquiterpenes, and momilactone diterpenes are allelopathic terpenes. Studies have shown that monoterpenes, citronellal, linalool, and cineole prolong the weed germination time and also reduce their development, e.g., *Cassia occidentalis*. Citronellal and linalool at 110 M and 55 M concentrations, respectively, completely stifled weed germination [112]. A related study on *Raphanus sativus L.* and *Lepidium sativum*, geraniol, carvone, borneol, and citronellol conferred significant inhibitory effects on seed sprouting and radical elongation of the two species of plants [113,114]. Nishida et al. also reported that 1,8-cineole, camphor, camphene beta-pinene, and alpha-pinene monoterpenes stifled plant cell proliferation and meristematic DNA synthesis. 

Some sesquiterpenes also exhibit allelopathy. Arbuscular A, achillin, viscidulin C, caryophyllene, bisabolone, and chamazulene play inhibitory functions. β-caryophyllene extract from the roots and pines of *Pinus* *halepensis* inhibited herbaceous plant growth [115].

Several studies on allelopathy elicitation and its effects have been reported. In the USA and Egypt, 557 out of 17,000 and 45 out of 1000 rice accessions, respectively, evaluated, conferred inhibitory effects on weed growth. Momilactone diterpenoids were confirmed as the inhibitors. For example, *Lepidium sativum* (barnyard grass) and *Cyperus difformis* L (rice sedge) were inhibited by constitutively synthesized momilactone B in rice roots [116,117]. Arabidopsis germination was also hampered by Momilactone A and B at concentrations higher than 30 and 10 *μ*M and IC_50_ 742 and 48.4 *μ*M, respectively. At 4, 20, 20 ppm, momilactone B completely stifled the sprouting of *Leptochloa chinenesis*, *Amaranthus retroflexus*, and *Cyperus difform*, respectively [68,118]. At varying concentrations of momilactone B, the growth and development of several monocots and dicots plants, including *Lepidium sativum* (cress), *Brassica rapa* cv, Chinese cabbage, *Lactusa sativa* cv, Santanasu (*lettuce*), *Echinochloa colonum* crabgrass, and *Phleum pretense* (timothy), among others, were also inhibited [119,120]. Momilactone inhibitory function in barnyard grass is linked to miRNA expression changes, which act as a hormonal signal transducer, DNA repairs by nucleotide excision, and the peroxisome proliferator-activated receptor pathway (PPAR pathway), and the p53 signaling pathway [120]. In another study, when momilactone A or B were sprayed on Arabidopsis, cruciferin 2 and 3 and cruciferina proteins responsible for providing the primary nitrogen for seed germination were highly expressed. Momilactone inhibiting cruciferins and cruciferina degradation might have consequently inhibited the germination of the Arabidopsis seeds. Moreover, the accumulation of amyrin synthase LUP2, *β*-glucosidase, a subtilisin-like serine protease, and malate synthase after momilactone treatment resulted in inhibited germination of Arabidopsis [118]. Allelopathy is illustrated in Figure 5c and Table 3.

**Table 1:** Insecticidal Activities of Terpenes.

Name of Terpene	Plant	Activity	Reference
Eucalyptol	Oak	Attracts cockchafer larva	[121]
Rhizathalene A	Arabidopsis	Resistance of the roots to the herbivore dark-winged fungus gnat (*Bradysia* spp)	[122]
Eugenol, caryophyllene oxide, α-pinene, α-humulene, and α-phellandrene	Cinnamon and clove	Secret toxic terpenes to deter the adult pest of *Sitophilus granaries* (grain weevil)	[123]
β-Pinene	*Citrus paradisix*, *Poncirus trifoliata*	Attracts entomopathogenic nematodes, e.g., *Steinernema diaprepesi*	[124]
S-methyl methionine	Lecythidaceae	Deters oviposition sites-seeking beetle	[125]
1,8-cineole	Brassica Tropical orchids	Attracts egg-laying parasitoids to the caterpillars of feeding herbivores. Furthermore, attracts and reward pollinators	[126]
β-trans-ocimene, (+)-R-limonene	Lavender	Deters pests, e.g., aphids	[127]
β-costic acid	*Zea mays*	Inhibits the growth of *Diabrotica balteata* (cucumber beetle)	[128]
β-ocimene	Tomato and tobacco	Defense against pests, e.g., *Macrosiphum euphorbiae* (potato aphid)	[129]
Decanal	*Zea mays*	Enhanced resistance against *Ostrinia nubilalis*	[130]

**Table 2:** Antimicrobial (Phytoalexin) Activities of Terpenes.

Name of Terpene	Plant	Activity	Reference
α-Terpinene Terpinen-4-ol α-Thujene	*Oryza sativa*	Antibacterial activity on Xoo	[131,132]
Linalool	*Oryza sativa*	Antibacterial activity on Xoo	[133]
epoxydolabranol	*Zea mays*	Defense against *F. verticillioides* and *F. graminearum*	[101]
Kauralexin A3 and B3	*Zea mays*	Antifungal activity against *R. microsporus* and *C. graminicola*	[134]
9β-Pimara-7,15-diene-3β,6β,19-triol	Rice leaves	Weak antimicrobial	[99]
Marneral	*A. thaliana*	Pathogenesis activities	[135]
Cucurbitadieno	*Cucumis sativus*	Pathogenesis and insecticidal activities	[136]
Oryzalexin A–F	*Oryza sativa* *Leersia perrier*	Antimicrobial (Inhibits spore germination and the growth of the germ tube of *O. oryzae*)	[24,99,136]
Phytocassane A–F	*Oryza sativa*	Antifungal activities *M. oryzae* and *R. Solani*	[99]
Oryzalexin S	*Oryza sativa*	Antifungal activity	[137]
Momilactone A, momilactone B,	*Oryza sativa*	Antimicrobial activities	[138]
Tirucalla-7, 24-dien-3b-ol	*A. thaliana*	Pathogenesis-related activities	[139]

**Table 3:** Allelopathic Activities of Terpenes in Agriculture.

Name of Terpene	Plant	Activity	Reference
Momilactone A	*Oryza sativa*, *Arabidopsis*, alfalfa, lettuce, cress, timothy, barnyard grass, *E. colonum*, crabgrass	Momilactones are generally not toxic to rice plants but mainly inhibit germination and growth of other seeds, e.g., barnyard grass Enhances plants to outcompete other field crops by roots exudates serving as inhabitants, especially Momilactones A and B	[120,140,141,142]
Momilactone B			
Momilactone C			
Momilactone D			
Momilactone E			
Carvone, Betulin	*Amaranthus retroflexu, Sinapis arvensis*	Exhibits high inhibition against common weeds at a lower concentration	[143]
Limonene and (+)-citronellal	Transgenic *Arabidopsis thaliana*	Exhibits vigorous antimicrotubule activity in transgenic *Arabidopsis thaliana*	[144]
Lanast-7,9(11)-dien-3α, 15α-diol-3α-D-glucofuranoside	*Oryza sativa*	Growth inhibition	[145]

## 4. Other Applications of Terpenes 

Apart from the role of plant protection, terpenes also offer a wide range of prospects in the pharmaceutical, food, cosmetic, and flavoring industries.

In the petrochemical industries, volatile terpenes are being explored as emerging alternatives for energy production. Leveraging plant-derived terpenes as an alternative energy source is a sustainable way of alleviating the over-dependence on fossil fuels and their accompanying adverse impact on atmospheric CO_2_ and climate change [146]. Hellier et al. reported that terpenes could be a fuel source in isolation or a blend of 65% in gasoline or diesel machines. Harvey et al. also suggested that hydrogenated valencene, premnaspirodiene, and caryophyllene could be used to generate high-density fuels using heterogeneous acid catalysts, Nafion SAC-13 [147,148,149]. 

Terpenes are used as flavoring and fragrance compounds in the food and toiletries industries, contributing over 5.3 billion USD annually in the United States, with a 3.7% increase in annual demand [150]. For example, (-)-Menthol (1-menthol) extracted from *Mentha arvensis* (Wild mint) is a flavoring ingredient in pharmaceuticals, cigarettes, cosmetics, chewing gums, and toothpaste manufacturing [150,151]. Menthone and its stereoisomers (menthone and isomenthone), extracted from *Pelargonium geranium*, are added to beverages to enhance their cooling, misty, and sweet scent (Geraniums) [152]. The flavor and fragrant properties of linalool make it a valuable ingredient in food and drinks, perfumes, and cosmetic products [150].

Isoprenoid resources remain unexhausted natural products, as many research investigations are still unearthing their beneficial values as antidotes to human problems. There has been a consistent molecular and pharmacological analysis of terpenes for potential anti-inflammatory, anti-tumor, anti-oxidative, antiaggregatory, and anti-coagulative effects. Some terpenoids exhibited highly extreme activity against malaria and cancer. For example, the anticancer property of Taxol^®^, a drug obtained from diterpenoids, and artemisinin obtained from a sesquiterpene, lactone, are among the vital pharmacological products from terpenes that are still effective to date. Terpenes confer anticancer activity by inducing apoptosis or necrosis to inhibit tumor cell proliferation in isolation or a blend with chemotherapy substances, e.g., β-Caryophyllene Eugenol, Menthol, limonene, and Ingenol 3-angelate (the diterpene), among others [32,153,154]. 

Despite the massive application of terpenes, a few factors, however, impede the realization of their full potential as the much-desired alternatives to synthetic compounds. The commercial quantity of natural plant-derived terpenes is minute and primarily does not meet commercial quantities. Microbes remain an excellent alternative as bio-factories for their engineering. However, the higher titers of some terpenes pose another survival limitation for the microbes. That notwithstanding, the prospects of sophisticated omic tools, such as DNA and RNA sequencing tools, Proteomics, CRISPR/Cas9, genome editing, and metabolic profiling tools, can be employed to engineer microbes that tolerate higher concentrations of these terpenes to meet commercial quantities.

## 5. Conclusions

Plants are not motile; hence, they cannot escape from their enemies. However, nature duly compensated for their immobility with the ability to produce several secondary metabolites, including terpenes that enhance their defense against microbial pathogens, insect pests, and weeds. The high incidence of pests and diseases resistance to synthetic pesticides calls for swift attention switch to terpenes and other plant-derived metabolites that offer limitless potentials with fewer or unknown hazards to human health and the environment.

## Abbreviations

PSM	Plants Secondary Metabolite
IPP	Isopentenyl diphosphate
DMAPP	Dimethylallyl diphosphate
MVA	Mevalonate pathway
MEP	2C-methyl-d-erythritol-4-phosphate
GPP	Geranyl pyrophosphate
FGPP	Farnesyl geranyl pyrophosphate
GGPP	Geranylgeranyl pyrophosphate
GPPS	Geranyl pyrophosphate synthase
FPPS	Farnesyl pyrophosphate synthase
TPS	terpenes synthase
NGS	Next-Generation Sequencing
IPPC	International Plant Protection Convention
Xoo	*Xanthomonas oryzae pv. oryzae*
EOs	Essential oils
PPAR pathway	Peroxisome proliferator-activated receptor pathway
DXP	1-deoxy-d-xylulose 5-phosphate
CDP-ME	4-diphosphocytidyl-2-C-methyl-d-erythritol
CDP-MEP	4-diphosphocytidyl-2-C-methyl-d-erythritol-2-phosphate
MEcPP	2-C-methyl-d-erythritol-2,4-cyclodiphosphate
HMBPP	4-hydroxy-3-methyl-butenyl 1-diphosphate
CTP	cytidine 5’-triphosphate
IDI	isopentenyl-pyrophosphate delta isomerase
HMG-CoA	3-Hydroxy-3-methylglutaryl-coenzyme A
CTP	Cytidine triphosphate
